# Tamarix honey phenolics attenuate cisplatin-induced kidney toxicity by inhibition of inflammation mediated IL-6/STAT3/TNF-α and oxidative stress-dependent Nrf2/caspase-3 apoptotic signaling pathways

**DOI:** 10.3389/fphar.2025.1584832

**Published:** 2025-07-04

**Authors:** Hanan Aati, Sultan Y. Aati, Hebatallah S. Bahr, Asmaa Ramadan Abdel-Sattar, Marwa Ahmed Embaby, Ahmed M. Reda, Usama Ramadan Abdelmohsen, Gerhard Bringmann, Hossam M. Hassan, Mostafa A. Darwish

**Affiliations:** ^1^ Department of Pharmacognosy, College of Pharmacy, King Saud University, Riyadh, Saudi Arabia; ^2^ Dental Health Department, College of Applied Medical Sciences, King Saud University, Riyadh, Saudi Arabia; ^3^ Department of Pharmacognosy, Faculty of Pharmacy, Nahda University, Beni-Suef, Egypt; ^4^ Department of Pharmacology and toxicology, Faculty of Pharmacy, Nile Valley University Egypt, Fayoum, Egypt; ^5^ Department of Medical Biochemistry, Faculty of Medicine, Beni-Suef University, Beni-Suef, Egypt; ^6^ Department of Pharmacy, Kut University College, Al Kut, Iraq; ^7^ Department of Biochemistry, Faculty of Pharmacy, Egyptian Russian University, Badr, Cairo, Egypt; ^8^ Deraya Center for Scientific Research, Deraya University, Minia, Egypt; ^9^ Department of Pharmacognosy, Faculty of Pharmacy, Minia University, Minia, Egypt; ^10^ Institute of Organic Chemistry, University of Würzburg, Am Hubland, Würzburg, Germany; ^11^ Department of Pharmacognosy, Faculty of Pharmacy, Beni-Suef University, Beni-Suef, Egypt; ^12^ Department of Pharmacology and Toxicology, Faculty of Pharmacy, Sphinx University, Assuit, Egypt

**Keywords:** cisplatin, nephrotoxicity, caspase-3, Nrf2, oxidative stress, STAT3 cisplatin, stat3

## Abstract

**Introduction:**

Cisplatin (CIS) is a productive chemotherapeutic agent that is effective against a variety of cancer types. Its utilization is linked to acute kidney injury and other adverse consequences. Among its toxic effects are oxidative stress, apoptosis as well as inflammation. Saudi Tamarix honey (STH) is a valuable product with plentiful nutritional and health benefits, demonstrating advantageous effects against inflammation and oxidative stress. Therefore, this study examined the potential of STH to prevent oxidative stress, apoptosis, inflammation, and kidney impairment that are induced by CIS in rats, pointing to the entanglement of the Nrf2, the caspase-3, and the IL-6/STAT3/TNF-α signaling pathways.

**Method:**

Histopathological examinations of the kidney were also used to evaluate cisplatin-induced nephrotoxicity. The rats received STH (50, 100 mg/kg) for 10 days and were challenged with a single dose of CIS (7 mg/kg) on day 7.

**Results:**

CIS caused injury to the glomeruli and the tubules, increased lipid peroxidation, TNF-α, IL-6, cleaved caspase-3, and decreased cellular antioxidants in the kidneys of rats. STH effectively prevented tissue injury, and ameliorated oxidative stress, inflammatory markers, in addition to caspase-3 in CIS-administered rats. STH is rich with antioxidants, suppressed STAT3 protein expression, and upregulated Nrf2 in CIS-administered rats. In conclusion, STH mitigated CIS-induced kidney injury by reducing oxidative stress, suppressing STAT3 and caspase-3, inhibiting pro-inflammatory mediators, and enhancing Nrf2 signaling. On the other hand, metabolomic profiling proposed the presence of 15 metabolites belonging to the chemical classes, phenolic acids, flavonoids and sterols, where phenolic acids were the most abundant classes.

## 1 Introduction

Cisplatin (CIS) is a highly effective and extensively used anti-neoplastic drug worldwide. It is employed to counteract a diverse array of cancers, such as those that affect the genitalia, ovaries, bladder, cervical region, lung as well as head and neck (Darwish et al., 2018). Unfortunately, CIS is restricted in its clinical use due to the numerous toxicities it elicits, such as nephrotoxicity, hepatotoxicity, cardiotoxicity, neurotoxicity, and ototoxicity. Although it has a practical applicability as an anti-neoplastic drug, these toxicities diminish the value of its clinical use (Darwish et al., 2017; Neamatallah et al., 2018; Hassan et al., 2020). There is a substantial body of experimental research that has demonstrated the widespread toxic side effects of CIS, with nephrotoxicity being the most notable one (Tahoon, 2017). The toxicity induced by CIS is driven by the production of reactive oxygen species (ROS), which results in elevation in cell injury and lipid peroxidation. In the same way, cell arrest and apoptosis are outcomes of an excess of reactive oxygen species in both non-target normal cells and cancer cells (Galadari et al., 2017; Palipoch et al., 2014). While the primary mechanisms of toxicity induced by CIS are the increased oxidative stress and inflammation, it is necessary to uncover additional molecular pathways. The regulation of physiological processes that serve to inhibit the development and progression of CIS-induced renal injury has been suggested by a multitude of studies. Nuclear E2-related factor 2 (Nrf2) is one such factor (Zúñiga-Toalá et al., 2013; Polat et al., 2018). The pharmacological activation of Nrf2 has been observed to inhibit CIS-mediated nephrotoxicity, whereas the absence of Nrf2 has been reported to exacerbate CIS-induced nephrotoxicity (Li et al., 2018). The pharmacological activation of Nrf2 is, thus, regarded as a critical molecular target for the prevention of acute kidney injury (AKI) induced by CIS. Furthermore, the signal transducer and activator of transcription 3 (STAT3) functions as a transcription factor and signal mediator in cancer and inflammation (Yu et al., 2009). STAT3 upregulation was observed in ischemia-reperfusion and CIS-induced nephrotoxicity, whereas STAT3 repression exhibited a nephroprotective effect (Peng et al., 2016).

Recently, there has been a growing focus on the protective abilities of natural substances that possess anti-inflammatory and antioxidant properties, and on the mechanism(s) through which they conduct their effects. In the past decade, there has been a significant increase in the use of natural products and dietary antioxidants to safeguard against toxicity caused by CIS. *Tamarix* sp., widely known as tamarisks, which mainly contain polyphenols as their secondary metabolites, can show promising anti-inflammatory and antioxidants properties (Rasouli et al., 2017). *Tamarix* sp. has been shown to have multi-pharmacological activities such as anti-oxidant, anti-inflammatory, antidiabetic, anticancer, chemoprevention, and anti-Alzheimer effects (Bahramsoltani et al., 2020).

The high efficacy demonstrated by STH use in protecting against oxidative damage and inflammation raises the possibility of its potential as an adjuvant in both the treatment and avoidance of a wide range of medical disorders. Nevertheless, no research has so far been conducted to ascertain the protective effect of STH against the CIS-induced nephrotoxicity. Consequently, the current study examined the potential nephroprotective effects of STH on oxidative stress, apoptosis, and inflammation, as well as its impact on the inhibition of the Nrf2/caspase-3 and IL-6/STAT3/TNF-α signaling pathways in CIS-induced nephrotoxicity, at two distinct doses. Additionally, STH metabolic profiling was investigated using LC-HRMS in order to identify its chemical metabolites.

## 2 Materials and methods

### 2.1 Chemicals, kits, and antibodies

The honey sample was supplied from a store specialized in selling high-quality honey (Mountain’s Honey Store, Riyadh). It had been collected from *Tamarix aphylla* L. (Tamaricaceae), a tree growing in the Fayfa Mountains of the southern region, Kingdom of Saudi Arabia (KSA), in 2022. Cisplatin was purchased from Mylan SAS pharmaceutical company (Saint-Priest, France). Kits for reduced glutathione (GSH) (CAT No. GR2511), malondialdehyde (MDA) (CAT No. MD2529), total antioxidant capacity (TAC) and superoxide dismutase (SOD) were purchased from Biodiagnostic (Cairo, Egypt). Nuclear factor erythroid-derived 2-like 2 (Nrf2, CAT No. YPA 1865), cleaved caspase-3 (CAT No. YPA2210) polyclonal antibodies, and β-actin monoclonal antibody (CAT No. BTL1027) were obtained from Biospes (Chongqing, P.R. China), while STAT3 monoclonal antibody (CAT No. SC-293151) was purchased from Santa Cruz Biotechnology (Texas, United States). Rat ELISA Kits of TNF-α (CAT No. E-EL-R0019) and interleukin 6 (IL-6) (CAT No. E-EL-R0015) were obtained from Elabscience Biotechnology (Texas, United States).

### 2.2 LC-HRMS metabolomic analysis

The metabolic analysis of Saudi Tamarix honey extract was performed using LC-HR-ESI-MS, as previously described by Abdelmohsen et al. (2014). A 1 mg/mL ethyl acetate soluble fraction in MeOH was injected and analyzed using an Accela HPLC system (Thermo Fisher Scientific, Karlsruhe, Germany) coupled with a UV-visible detector and QTOF instrument [Agilent 6500 Series Q-TOF], using an HPLC column (an ACE C18, 75 mm × 3.0 mm, 5 μ column (Hichrom Limited, Reading, UK). Purified water (A) and acetonitrile (B) with 0.1% formic acid in each mobile phase were used for the gradient elution, which was performed at 300 μL min-1 for 30 min. The gradient program began with 10% B, increased gradually to 100% B, and then continued isocratic for 5 min before linearly decreasing back to 10% B for 1 min. The total analysis period for each fraction was 45 min. The column temperature was kept at 20°C, and the injection volume was 10 μL. In order to cover a large number of metabolites, high-resolution mass spectrometry was performed using positive and negative ESI ionization modes in conjunction with a spray voltage of 4.5 kV, capillary temperature of 320°C, and a mass range of m/z 150 to 1500. The data mining program Mzmine 2.10 (Okinawa Institute of Science and Technology Graduate University, Japan) was used to treat the acquired MS data for deconvolution, peak picking, alignment, deisotoping, and molecular formula prediction before dereplication. The structures of the chemical formulas were drawn using the ChemBioDraw Ultra 14.0 software.

### 2.3 Experimental animals

All study protocols involving animals were approved by the Institutional Animal Care and Ethical Committee at Nahda University in Beni-Suef, Egypt (NUB-025–037). Adult male Wistar albino rats (Animal Facility, Faculty of Pharmacy, Nahda University, weighing 180–220 g) were fed standard laboratory chow (El-Nasr Company, Abou-Zaabal, Cairo, Egypt) and water *ad libitum*. Rats were accommodated at 22°C ± 2°C with humidity of 50% ± 10% with 12-h dark-light cycle.

### 2.4 Acute-toxicity study

The “up-and-down” test method was employed to assess the acute oral toxicity of the STH in rats at a single dose of 2000 mg/kg, in accordance with the limit test recommendations of the Organization for Economic Development (OECD) No. 425 Guideline (Bruce, 1987; Zarei et al., 2023). In our investigation, six Wistar albino rodents were employed for each dose. One rat was administered 2000 mg/kg of STH extract orally on the first day of the experiment after fasting for 3 h. The rat was subsequently observed individually at least once during the initial 30 min and on a regular basis for the subsequent 24 h, with a particular emphasis on the first 4 h. The first rat did not exhibit any mortality; consequently, the additional five fasting rats were sequentially administered a single dose of STH (2000 mg/kg) and subsequently monitored for 14 days for any indications of toxicity or mortality. Various symptoms like salivation, lethargy, diarrhea, respiratory distress, convulsions, tremor, and sleeping were meticulously recorded.

### 2.5 Experimental design

The rats were randomly assigned to four groups, each consisting of six animals, following a 2-week acclimatization period.

- The control group: these animals were provided with a vehicle for a period of 10 days.
- The CIS group received a single intraperitoneal dosage of 7 mg/kg CIS on the seventh day of the study. - The CIS +50 mg/kg honey group: the rats in this group received a single intraperitoneally (i.p.) dose of 7 mg/kg CIS on the seventh day of the experiment, in addition to 50 mg/kg honey administered once daily for 10 days.
- The CIS +100 honey group: the rats were administered 100 mg/kg honey intraperitoneally (i.p.) once daily for a period of 10 days, in conjunction with a single i.p. administration of 7 mg/kg CIS on the seventh day of the experiment.

The dose and experimental design of STH and CIS administration were guided by previous work (Fathy et al., 2022; Atwa et al., 2022).

### 2.6 Serum and tissue samples collection

After 10 days of experiment, the rats were anesthetized with diethyl ether and euthanized by cervical dislocation. The kidney tissue samples that were collected were divided into two-halves. One-half was fixed in 10% formalin for histopathological analysis. The other half was frozen in liquid nitrogen and stored at −80°C for biochemical, Elisa, and Western blot analysis. Blood samples were obtained from the retro-orbital plexus using small capillary tubes prior to sacrifice. In a refrigerated centrifuge, serum was prepared from blood by centrifugation at 3000 rpm for 10 min. The serum samples were subsequently stored at −80°C for further biochemical analysis.

### 2.7 Biochemical assays

Blood samples, freshly collected after scarification were centrifuged at 3000 rpm for 10 min. The obtained clear serum was used for the analysis of biochemical parameters, like blood urea nitrogen (BUN) and creatinine, using commercially available colorimetric assay kits (Sigma-Aldrich), according to the standard procedures. Urine was collected on day 10, by keeping the animals in individual metabolic cages.

### 2.8 Lipid peroxidation and antioxidant status evaluation

In accordance with the manufacturer’s instructions, commercially available kits (Biodiagnostic, Egypt) were employed to evaluate the levels of MDA, GSH, TAC, and SOD activity in the kidney homogenates.

### 2.9 Anti-inflammatory status evaluation

The evaluation of TNF-α and IL-6 levels in the kidney homogenates was conducted in accordance with the manufacturer’s instructions using commercially available Elisa kits from Elabscience Biotechnology (Texas, United States).

### 2.10 Histopathological examination

At the end of the experiment, the kidneys were obtained and fixed in 10% neutral buffered formalin. They were routinely dehydrated in ethanol and embedded in paraffin wax. This was followed by the sectioning of the blocks at a thickness of 4–5 mm. Tissue segments were collected on clean, dry glass slides, deparaffinized, and stained with hematoxylin and eosin (H&E) (Bancroft and Gamble, 2008). Each slide were then investigated and visualized for pathological changes using a light microscope at ×200 magnification (Leika DMRBE, Germany).

### 2.11 Protein extraction and Western blot analyses

Western blots were conducted in accordance with the method previously described (Martin et al., 2007) Protein extraction was conducted using ready Prep™ buffer (Bio-Rad Inc., catalog #163–2086), which included the lysis of the kidneys of all experimental animal groups. The protein contents of the lysates were determined by centrifugation and assessed using a Bradford protein assay reagent. Subsequently, 20 µg of protein from each sample were combined with Laemmli loading buffer and separated by 10% sodium dodecyl sulfate–polyacrylamide electrophoresis. The isolated protein contents were then blotted on a nitrocellulose membrane (Millipore, Burlington, MA, United States). The membrane was incubated with primary antibodies against cleaved caspase-3, Nrf2, STAT3, and β-actin, after being blocked with 5% skim milk. Subsequently, secondary antibodies (HRP-conjugated goat IgG) were added. The enhanced chemiluminescent reagent was employed to visualize the proteins. The membrane was re-probed with β-actin to confirm that the individual sample proteins were loaded equally. Using densitometry, protein bands were semi-quantified in relation to β-actin, and the results were presented as a bar chart.

### 2.12 Statistical analysis

The data were analyzed using one-way analysis of variance (ANOVA) for multiple group comparisons, followed by a Tukey–Kramer *post hoc* test using GraphPad Prism, version 8 (CA, United States). The difference was considered significant at *p* < 0.05. The data were expressed as mean ± SEM.

## 3 Results

### 3.1 Metabolomic profile of Saudi Tamarix honey extract

**FIGURE 1:**
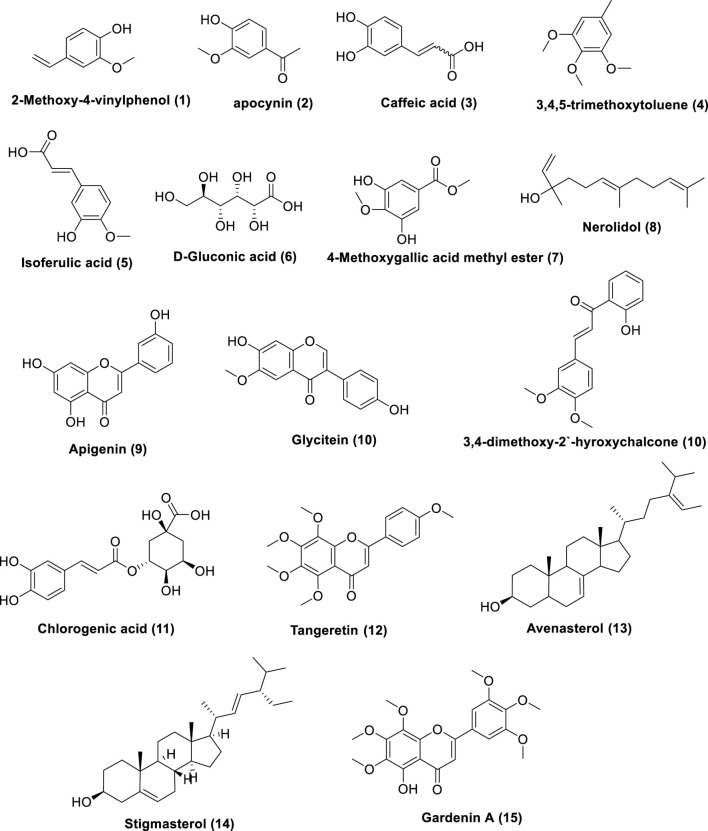
Metabolites dereplicated from Saudi Tamarix honey.

The prepared extract of Saudi Tamarix honey was analyzed in positive and negative ion mode by LC-HRMS; a total of 336 features were detected; 299 in positive and 37 in negative mode (Figure 1). Untargeted metabolomic approaches were performed to profile the metabolites present in the sample considering only low molecular weight (*m/z* < 1500) ionizable molecules. Dereplication was implemented using literature databases,the resulted features were reduced by applying a chemotaxonomic filter, which resulted in the identification of 15 metabolites (Table 1; Figure 1). The identified metabolites were mainly represented by phenols and phenolic acids.

**TABLE 1:** Metabolites dereplicated from Saudi Tamarix honey.

N	*m/z*	Rt	MW	Molecular formula	Identifcation	Source	Class	References
1	359.1247	2.569	150.0551	C_9_H_10_O_2_	2-Methoxy-4-vinylphenol	*Tamarix ramosissima Ledeb.* [Tamaricaceae]	Phenol	Xiao et al. (2013)
2	167.0123	2.056	166.0051	C_9_H_10_O_3_	Apocynin	*Tamarix ramosissima Ledeb.* [Tamaricaceae]	Phenol	
3	179.0585	2.657	180.0658	C_9_H_8_O_4_	Caffeic acid	*Tamarix aphylla (L.) H. Karst.* [Tamaricaceae] *Tamarix hispida Willd.* [Tamaricaceae]	Phenolic acid	Mahfoudhi et al. (2014)
4	183.08	22.66	182.0727	C_10_H_14_O_3_	3,4,5-Trimethoxytoluene	*Tamarix ramosissima Ledeb.* [Tamaricaceae]	Phenol	Xiao et al. (2013)
5	195.1021	23.56	194.0948	C_10_H_10_O_4_	*trans-*Isoferulic acid	*Tamarix aphylla (L.) H. Karst.* [Tamaricaceae]	Phenolic acid	Nawwar et al. (2009)
6	195.0541	2.439	196.0614	C_6_H_12_O_7_	D-Gluconic acid	*Tamarix aphylla (L.) H. Karst.* [Tamaricaceae]		Bahramsoltani et al. (2020)
7	215.0357	2.435	216.0429	C_9_H_12_O_6_	4-Methoxygallic acid methyl ester	*Tamarix hispida Willd.* [Tamaricaceae]	Phenolic acid	Sultanova et al. (2004)
8	245.0779	20.73	222.0886	C_15_H_26_O	*trans-*Nerolidol	*Tamarix boveana Bunge* [Tamaricaceae]	Sesquiterpene	Saidana et al. (2008)
9	269.0907	2.849	270.098	C_15_H_10_O_5_	Apigenin	*Tamarix aphylla (L.) H. Karst.* [Tamaricaceae] *Tamarix africana Poir.* [Tamaricaceae] *Tamarix ramosissima Ledeb.* [Tamaricaceae]	Flavonoid	Xiao et al. (2013) Mahfoudhi et al. (2014)
10	285.1255	9.615	284.1182	C_16_H_12_O_5_	Glycitein	*Tamarix ramosissima Ledeb.* [Tamaricaceae]	Flavonoid	(Xiao et al., 2013)
				C_17_H_16_O_4_	3,4-Dimethoxy- henol (3,4-dimethoxy-2′-hyroxychalcone		Flavonoid	
11	353.2181	29.37	354.2253	C_16_H_18_O_9_	*trans*-Chlorogenic acid	*Tamarix africana Poir. [*Tamaricaceae*]*	Phenolic acid	Chekroun-Bechlaghem et al. (2019)
12	373.1463	11.61	372.139	C_20_H_20_O_7_	Tangeretin	*Tamarix ramosissima Ledeb.* [Tamaricaceae]	Flavonoid	Xiao et al. (2013)
13	413.3178	34.55	412.3106	C_29_H_48_O	Avenasterol	*Tamarix gallica L.* [Tamaricaceae]	Sterol	Andhiwal et al. (1982)
14	413.3217	35.26	412.3144	C_29_H_48_O	Stigmasterol	*Tamarix gallica L.* [Tamaricaceae]	Sterol	
15	419.3144	39.26	418.3071	C_21_H_22_O_9_	Gardenin A	*Tamarix africana Poir.* [Tamaricaceae]	Flavonoid	Karker et al. (2016)

### 3.2 Biological study

The animals did not show mortality during the observation period in the acute toxicity investigation of STH at the limit dose of 2000 mg/kg. In addition, they did not exhibit any indications of restlessness, irritation, respiratory distress, diarrhea or convulsions. Consequently, the doses of STH that were investigated were considered a being harmless for rats.

#### 3.2.1 Effect of STH on renal biochemical parameters

**FIGURE 2:**
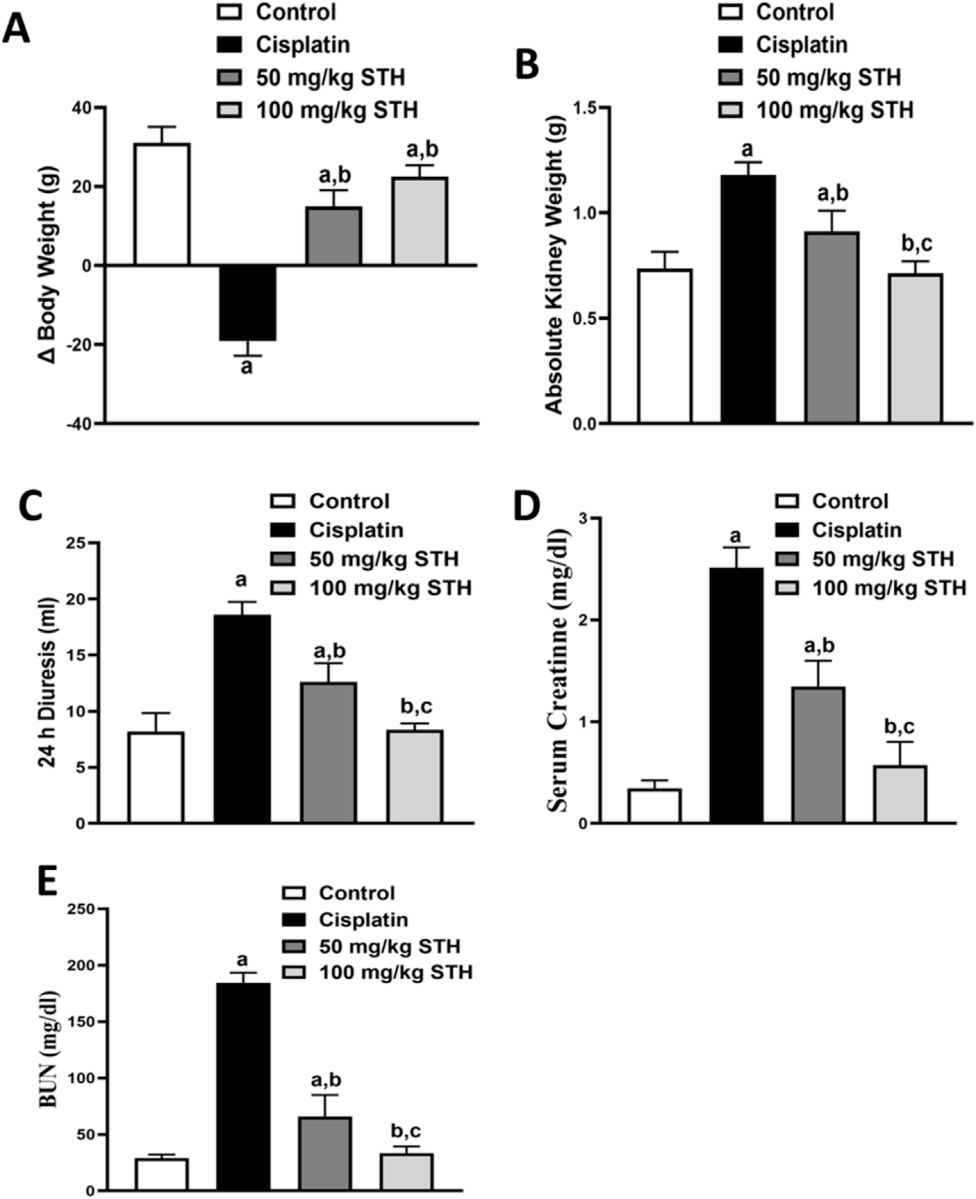
Effect of STH on kidney functions. Effect of different doses of STH on **(A)** difference in body weight; **(B)** absolute kidney weight, **(C)** 24 h diuresis; **(D)** serum creatinine; and **(E)** BUN. a Significantly different from the control group; b significantly different from the cisplatin group; and c significantly different from the STH 50 mg/kg group, using one-way ANOVA followed by Tukey-Kramer multiple comparison test at *p* < 0.05 (n = 6). ANOVA: analysis of variance; BUN: blood urea nitrogen; SEM: standard error of the mean; STH: Saudi Tamarix honey.

Saudi Tamarix honey (STH) was given in two doses (50 and 100 mg/kg) to get insight into the effect of the STH different doses on CIS-induced nephrotoxicity. CIS significantly increased absolute kidney weight, 24 h diuresis, serum creatinine, and blood urea nitrogen while markedly decreased difference in body weight as compared to the normal control group. Remarkably, the two doses of STH caused a significant attenuation in the aforementioned parameters in CIS-treated rats (Figures 2A–E).The equations should be inserted in editable format from the equation editor.

#### 3.2.2 Effect of STH on renal redox status

**FIGURE 3:**
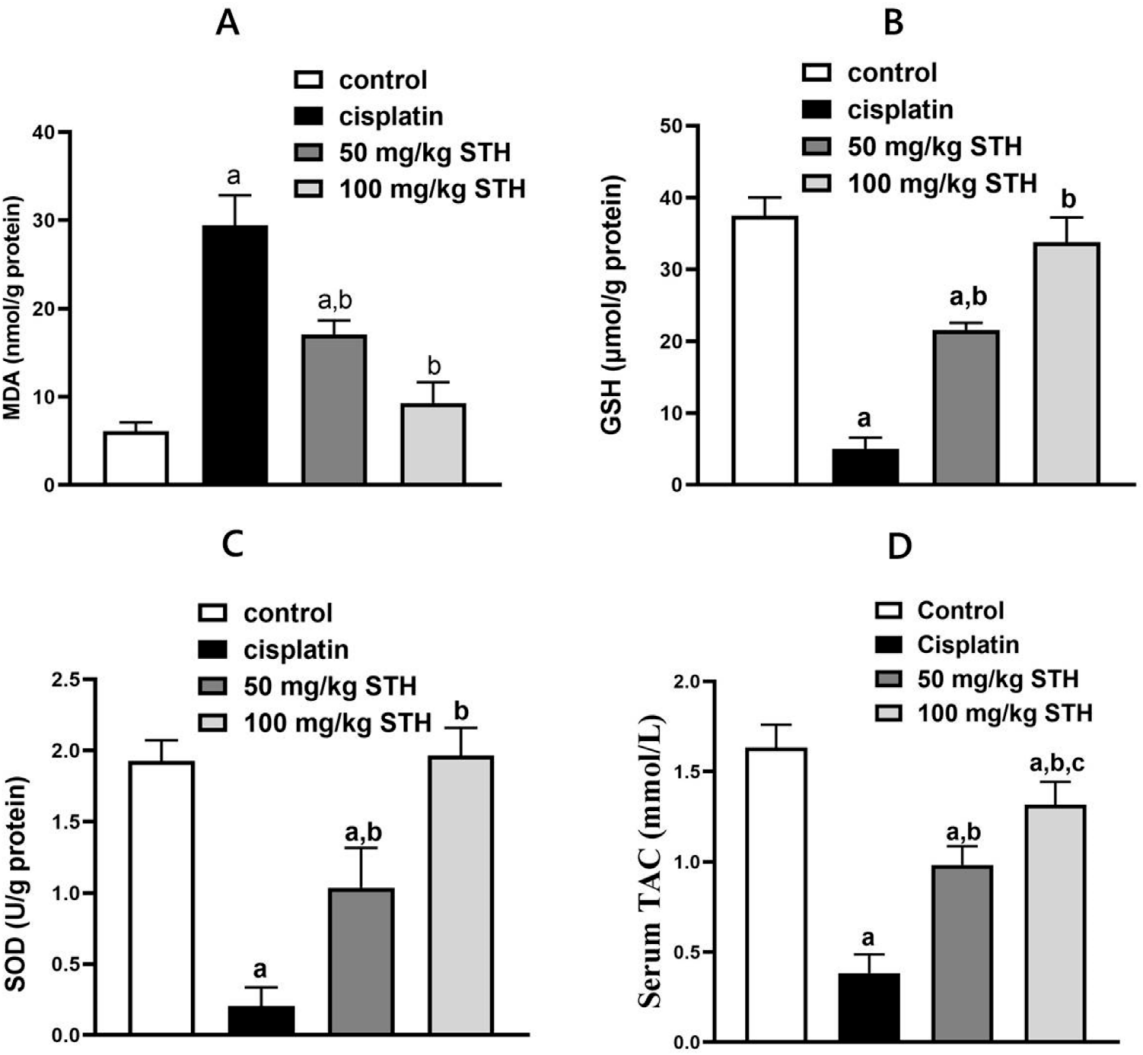
Effect of STH on the renal redox status. Effect of different doses of STH on renal **(A)** MDA, **(B)** GSH, **(C)** SOD, and **(D)** TAC. a Significantly different from the Control group; b significantly different from the Cisplatin group; c significantly different from the STH 50 mg/kg group, using one-way ANOVA followed by the Tukey-Kramer multiple comparison test at *p* < 0.05 (n = 6); MDA: malondialdehyde; GSH: glutathione; SOD: superoxide dismutase; TAC: total antioxidant capacity.

Oxidative stress was induced in the kidney of rats by the administration of CIS. In comparison to the control rats, it significantly increased the MDA content and significantly decreased the GSH content, SOD activity, and TAC. In contrast, the oxidative stress parameters induced by CIS in the rat kidney were notably ameliorated by STH in the two doses (50 mg/kg and 100 mg/kg). (Figures 3A–D).

#### 3.2.3 Effect of STH on the renal inflammatory status

**FIGURE 4:**
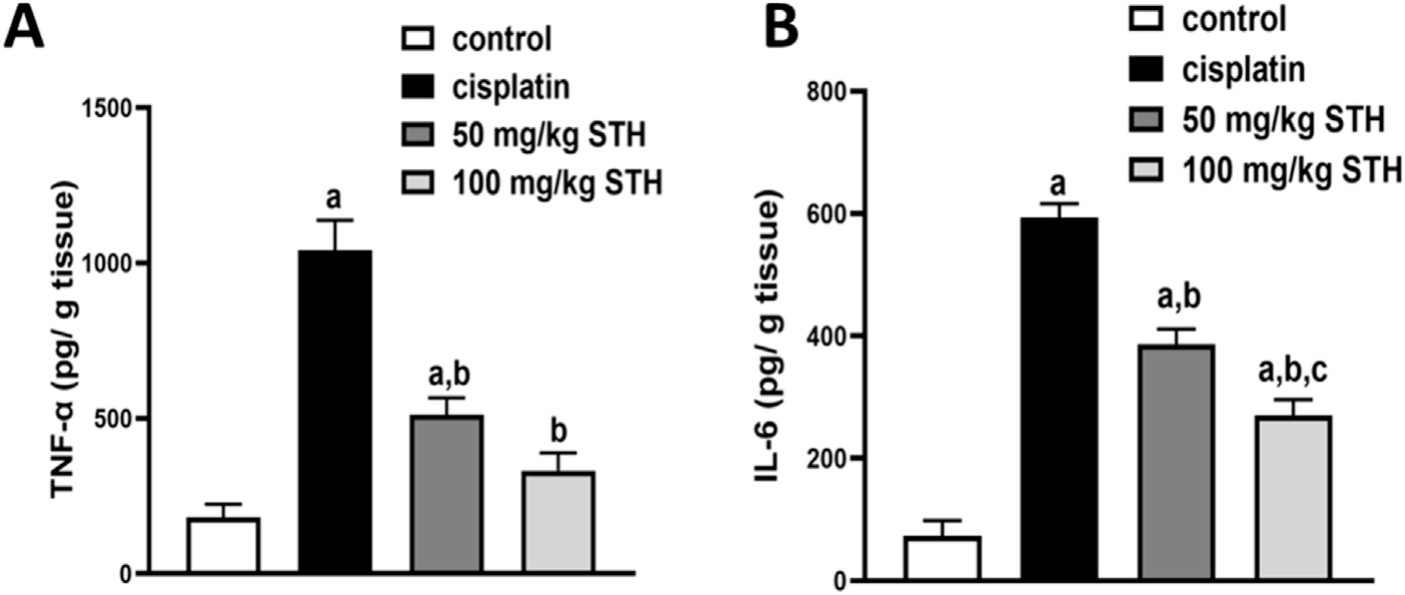
Effect on the renal **(A)** TNF-α and **(B)** IL-6 levels. Effect of different doses of STH on the renal TNF-α and IL-6 level. a Significantly different from the Control group; b significantly different from the Cisplatin group; c significantly different from the STH 50 mg/kg group, using one-way ANOVA followed by Tukey-Kramer multiple comparison test at *p* < 0.05 (n = 6). TNF-α: tumor necrosis factor-alpha; IL-6: interleukin-6.

Renal TNF-α and IL-6 concentrations were examined as inflammatory markers, which showed a significant increase in the CIS group when compared with the control group. Prior administration of STH in the two doses significantly reduced the renal TNF-α and IL-6 levels compared with the CIS group (Figures 4A, B).

#### 3.2.4 Effect of STH on renal histological features

**FIGURE 5:**
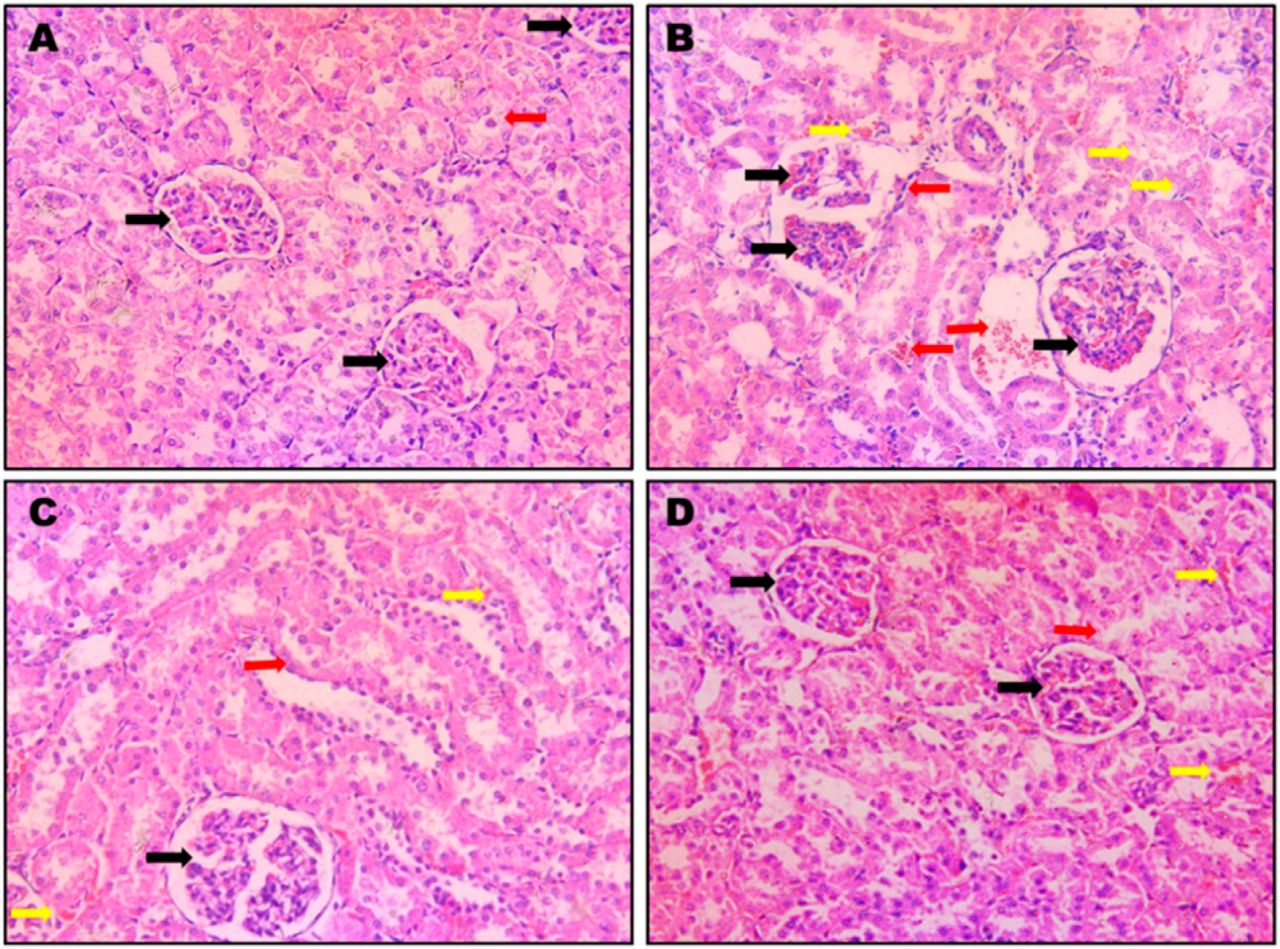
Photomicrographs of rat kidneys (H&E 200x). Effect of different doses of STH on renal histopathological changes. **(A)** A photomicrograph of a kidney section obtained from control rats; the section shows a normal renal architecture with normal corpuscles (black arrow) and convoluted tubules (red arrow); **(B)** a photomicrograph of kidney section obtained from CIS-treated (positive control) rats. The section displays bisected renal corpuscles (black arrow) and tubular congestion (yellow arrow) and interstitial edema and inflammations and immune cells infiltration (red arrow); **(C)** a photomicrograph of kidney section obtained from rats intoxicated with CIS and pre-treated with STH 50 mg/kg; the section shows a nearly normal structure of the kidney with normal renal corpuscles (black arrow), with most of the renal tubules being normal (red arrow) except for little interstitial hemorrhages (yellow arrows); **(D)** a photomicrograph of kidney section obtained from rats intoxicated with CIS and pre-treated with STH 100 mg/kg; the section shows a nearly normal structure of the kidney, with normal renal corpuscles (black arrows) and most of the renal tubules being normal (red arrow), except for little interstitial hemorrhages (yellow arrows).

The effects of different treatment modalities on normal kidney histological features are shown in Figure 5). CIS administration induced severe and widespread inflammation with congested bisected renal corpuscles and dilatation, as well as marked degenerated tubules, in addition to remarkable immune cells infiltrations. Administration of STH significantly improved the histopathological features in terms of existence and the severity of the damage (Figures 5A–D).

#### 3.2.5 Effect of STH on renal Nrf2, STAT3, and caspase-3 protein expression

**FIGURE 6:**
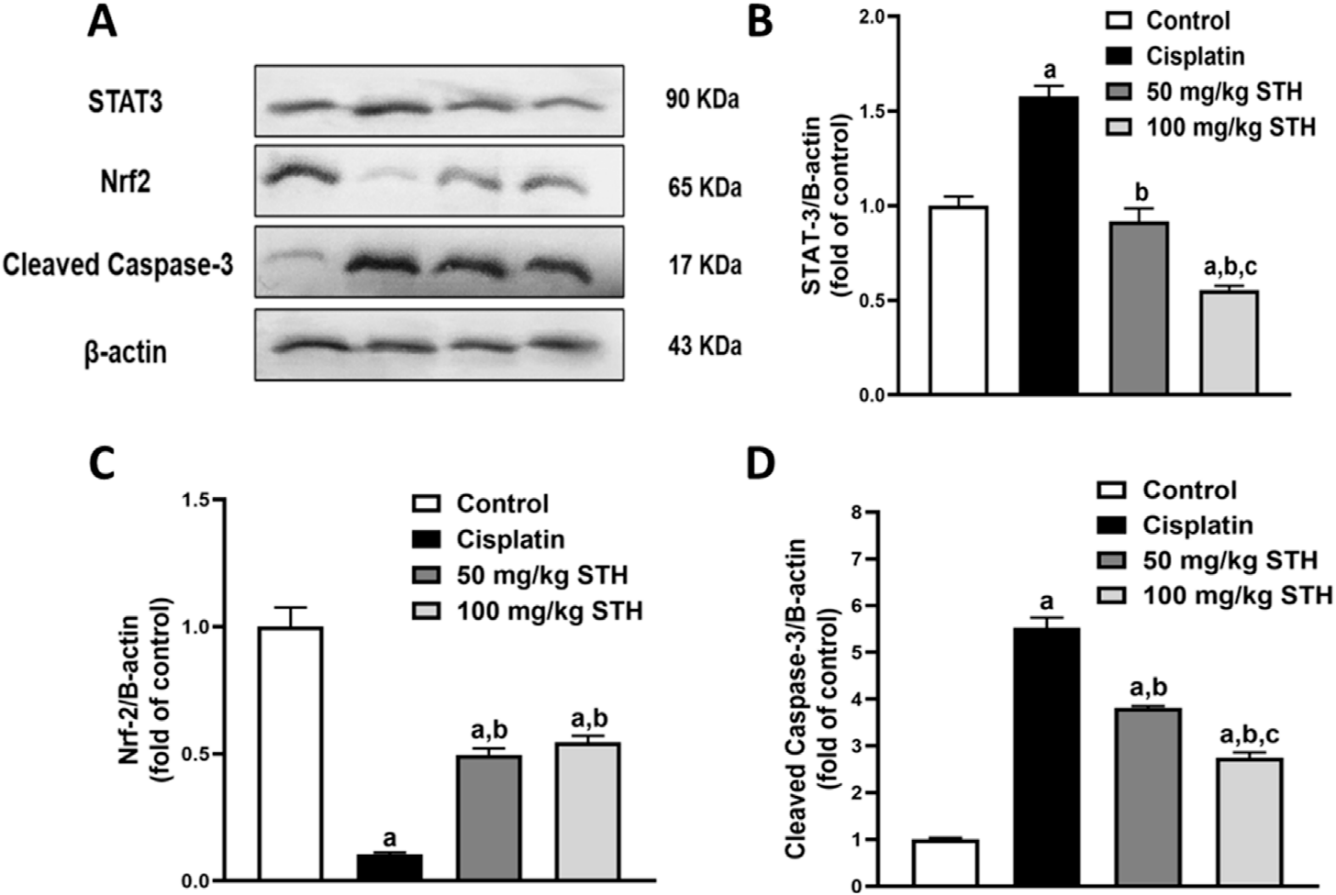
Effect of STH on renal STAT3, Nrf2, and caspase-3 protein expression. **(A)** Western blot analysis of kidneys from the normal group and the CIS group with or without STH using STAT3, Nrf2, and caspase-3 Abs together with β-Actin Ab as an internal control (n = 6). Quantitative analysis (fold changes) of western blots depicted effect of different doses of STH on **(B)** STAT3, **(C)** Nrf2, and **(D)** caspase-3 protein expression in graphs using ImageJ/NIH software. a Significantly different from the control group; b significantly different from the cisplatin group; c significantly different from the STH 50 mg/kg group using one-way ANOVA followed by Tukey-Kramer multiple comparison test at *p* < 0.05 (n = 6).

A significant downregulation of Nrf2 expression, but also a marked upregulation in STAT3 and caspase-3 protein expression as an apoptotic marker, in comparison to normal rats, was detected in the kidneys of CIS-injected animals, as demonstrated by Western blot analyses. Prior to CIS injection, the expression of Nrf2 was significantly elevated by the administration of 50 mg/kg and 100 mg/kg of STH, whilst, on the other hand, a profound decline was observed in STAT3 and caspase-3 protein expression in a dose-dependent manner when compared with the CIS-injected rats (Figures 6A–D).

## 4 Discussion

The current study assessed the protective effect of Saudi Tamarix honey (STH) in two distinct concentrations (50 mg/kg and 100 mg/kg) against CIS-induced toxicity in the kidney. This is the first report to examine the potential molecular protective mechanisms of STH in the context of CIS-induced nephrotoxicity, specifically its anti-inflammatory, antioxidant, and anti-apoptotic properties, which have not been previously evaluated, to the best of our knowledge.

Nephrotoxicity is a significant adverse effect of cisplatin, which is one of the most potent cytotoxic anticancer medications. This adverse effect is an obstacle to its use, as it has been associated with the excessive generation of ROS as well as cell and DNA damage (Dkhil et al., 2013). Furthermore, oxidative stress induces inflammation and triggers the release of cytokines such as TNF-α and IL-6 (Ingawale et al., 2014).

In this context, our study illustrates that CIS treatment leads to a severe array of nephrotoxic events, as evidenced by a substantial increase in serum creatinine, BUN, 24 h diuresis, and absolute kidney weight. Additionally, a significant decrease in body weight was observed, widespread inflammation with congested bisected renal corpuscles and dilatation, marked degenerated tubules in addition to remarkable immune cells infiltrations. The potential of these CIS-induced ameliorated function parameters may, in part, be due to a secondary event after CIS-induced kidney injury as shown in the histopathology investigation. In agreement with our results, previous investigations had reported an elevation in the serum creatinine, BUN, 24 h diuresis, absolute kidney weight, and declined change in body weight in CIS-induced nephrotoxicity (Darwish et al., 2017; Darwish et al., 2018; Ansari, 2017; Potočnjak et al., 2017; Zhang J. et al., 2020) Interestingly, concomitant treatment with STH (50 mg/kg and 100 mg/kg), however, restored the normal architecture of corpuscles and tubules as well as the aforementioned functional parameters of kidney in a dose-dependent manner. Side by side with our findings, abdel-wahab et al., (2017) showed that N-acetylcysteine (NAC), a potent antioxidant, also improved the biochemical markers related to kidney function (Abdel-Wahab et al., 2017).

Excessive ROS generation in rats treated with CIS corresponds to an imbalance of oxidant-antioxidant levels, which reduces their ability to scavenge ROS and promotes oxidative stress. In our findings, this is underscored by the increase in renal MDA content and the reduction of enzymatic antioxidants, such as renal SOD. Additionally, the renal tissue is rendered more susceptible to oxidative stress by the reduction of GSH levels in rats that have been induced by CIS. Moreover, the depletion of GSH levels in CIS-induced rats makes the renal tissue more susceptible to oxidative stress. Previous studies have already detailed the profound increase in renal MDA in addition to a notable decline in GSH level, TAC and SOD activity after CIS injection (Darwish et al., 2017; Abdel-Wahab et al., 2017; Darwish et al., 2018; Ansari, 2017) In contrast to the CIS group, the administration of STH in conjunction with CIS demonstrated protection against oxidative stress in the present study. The potential protection of STH against depletion of antioxidant enzymes and lipid peroxidation may disclose a plausible mechanism for the antioxidative action of STH. In a similar way, Sekkien et al. (2018) showed that *Tamarix senegalensis*, another *Tamarix* species, also exerted antioxidant properties, which decreased lipid peroxidation levels and enhanced concentrations of endogenous antioxidants, including GSH and catalase (Sekkien et al., 2018). It should be noted that in all oxidative-stress parameters, the higher dose of the extract (100 mg/kg) showed a better efficacy, similar to that of the negative control, in most parameters. Additionally, our findings are consistent with prior research that found *Tamarix gallica*, another *Tamarix* species, to be effective in augmenting *in-vivo* antioxidant defense mechanisms such as GSH level and superoxide dismutase activity in a hepatotoxicity model (Sehrawat and Sultana, 2006). Importantly, our findings are in parallel with previous research that found NAC to be effective in reducing redox parameters like MDA as well as enhancing *in-vivo* antioxidant defense mechanisms such as GSH level, TAC, SOD or catalase activities (Abdel-Wahab et al., 2017; Badr et al., 2023; Dickey et al., 2008; Elsayed et al., 2021).

Development of inflammatory response and the production of TNF-α have been reported to be stimulated by cisplatin-induced oxidative stress, which in turn might trigger a large network of pro-inflammatory cytokines, including IL-1β and IL-6 (Zhang et al., 2007; Neamatallah et al., 2018).

The current study predicted that renal oxidative stress, which was induced by a single dosage of CIS (7 mg/kg), would promote inflammation as demonstrated by a significant increase in TNF-α levels in renal tissue and a remarkable increase in IL-6 levels, which are inflammatory markers. More importantly, the concurrent administration of different STH doses significantly reduced this inflammatory response. Previous reports have already described the significant increase in renal TNF-α and IL-6 levels that results from CIS injection (Hassan et al., 2020; Atwa et al., 2022). In line with our findings, Sekkien et al. (2018) showed that *T. senegalensis*, yet another *Tamarix* species, also displayed anti-inflammatory activity by reduction of TNF-α (Sekkien et al., 2018). Likewise, the anti-inflammatory properties of STH are in parallel with previous research that found NAC to be effective in decreasing CIS-induced inflammatory signalling responses like TNF-α expression (Badr et al., 2023). The potential of the STH antioxidant action may be, in part, due to caffeic acid, found in STH after LC–HR–ESI–MS metabolic analysis, that was reported for his strong antioxidant and anti-inflammatory properties (Genaro-Mattos et al., 2015).

Since CIS-induced inflammatory responses in the kidneys involve multiple signaling pathways, we evaluated the changes in renal STAT3 protein expression in relation with IL-6 and TNF-α for further exploration of the mechanism(s) underlying the protective effect of STH.

STAT3 is a cytoplasmic transcription factor that is believed to be exert a crucial role in the host response to inflammation (Sun et al., 2019). In a previous study, STAT3 activation was reported in endotoxin-induced AKI in mice (Gao et al., 2013). Interestingly, cytokines, including TNF-α, have been found to trigger STAT3, resulting in the induction of pro-inflammatory signaling (Tu et al., 2012). Furthermore, another study suggested that STAT-3 activation may, in part, occur through IL-6 cytokines (Zhang F. et al., 2020). Similarly, Yang et al. (2017) stated that renal interstitial fibrosis, apoptosis, and inflammation in mice with unilateral urethral obstruction might be due to a p53-dependent increase in STAT3 expression (Yang et al., 2017). Most recently, upregulation of STAT3 was reported in cisplatin-induced AKI [33,39]. Importantly, in concordance with all these previous reports, our study revealed that CIS has significantly elevated STAT3 upregulation in kidneys of CIS-treated rats emphasizing its crucial role in both, inflammation and apoptosis. In our results we report, for the first time, that STH pretreatment significantly and dose-dependently attenuates STAT3 protein expression remarkably indicating the anti-inflammatory effect of STH that might be due to the inhibition of IL-6/STAT3/TNF-α signaling pathway in CIS-induced nephrotoxicity in CIS-treated rats.

The equilibrium between pro- and anti-apoptotic signals is shifted by cisplatin intoxication, which favors the pro-apoptotic cascade (Indran et al., 2011). TNF-α is a protein that is closely associated with apoptosis and is involved in inflammatory responses (Safhi et al., 2019). The activation of the NF-κB pathway is believed to be the cause of the oxidative stress induced by CIS, which resulted in apoptosis and inflammation (Neamatallah et al., 2018) as the interaction between oxidative stress and inflammation is a critical characteristic of cell death (Voltan et al., 2016). Likewise, the activation of initiator caspases, such as caspase-8 and caspase-9, during apoptosis leads to the activation of executioner caspases, such as caspase-3 and caspase-7 (Salvesen and Dixit, 1997). Moreover, the activation of cellular proteins and DNA fragmentation factors via executioner caspase-3 results in apoptosis-mediated changes (Tong et al., 2004). Furthermore, a growing body of evidence has demonstrated that the Nrf2 signaling pathway can significantly ameliorate CIS-mediated cell and tissue toxicity (Mirzaei et al., 2021).

To further decode the mechanism(s) underlying the protective effect of STH against CIS-induced apoptosis in the kidneys, we delved deeper into the molecular level and evaluated the changes in Nrf2 and caspase-3 molecular proteins expression by Western blotting.

Nrf2 is found in the cytoplasm as an inactive complex with Keap1, and it elicits cytoprotective and antiapoptotic activities. The expression of the HO-1 gene is activated by Nrf2 in the nucleus, which subsequently reduces cellular injury caused by oxidative stress (Polat et al., 2018; Zúñiga-Toalá et al., 2013; Li et al., 2018). The pharmacological activation of Nrf2 activation has been observed pharmacologically to inhibit CIS-mediated nephrotoxicity, while the absence of Nrf2 has been reported to aggravate nephrotoxicity induced by CIS. The expression levels of Nrf2, a critical protein that is involved in the regulation of antioxidant proteins and the inhibition of cell apoptosis, were assessed in the context of CIS and STH treatment. In the present study, 50 mg/kg STH and 100 mg/kg STH pretreatments showed, for the first time, a significant reduction in the apoptotic ability of CIS in kidneys. The results revealed that CIS downregulated the protein expression of Nrf2 while, on the other hand, remarkably upregulated cleaved caspase-3 protein expression. Intriguingly, STH promoted the expression of Nrf2, while it declined protein expression of cleaved caspase-3 in a dose-dependent manner, which suggested that STH may inhibit the apoptosis of kidneys and subsequently protect the kidney against CIS-induced renal injury. We can thus assume that inhibition of apoptosis pathways like cleaved caspase-3, Nrf2, and TNF-α can serve as a therapeutic intervention to prevent renal injury caused by cisplatin. Moreover, our results confirmed that the significant increase of cleaved caspase-3 and profound decrease in Nrf2 caused by cisplatin were completely ameliorated in STH-treated rats in a dose-dependent manner.

Taken together, the results of the current study suggest that administration of STH ameliorates nephrotoxicity induced by CIS. For the first time in the literature, we have thereby unraveled the protective effects of STH, not only for its antioxidant properties, but also for anti-apoptotic and anti-inflammatory activities coinciding via inhibition of Nrf2/caspase-3 and IL-6/STAT3/TNF-α signaling pathways. STH, especially the 100 mg/kg dose, could be considered as a renoprotective natural compound in CIS-induced kidney injury. Further studies are required to emphasize its beneficial effects in clinical trials.

### 4.1 Metabolomic profile of Saudi Tamarix honey

Metabolomics, the thorough study of small molecule metabolites in biological systems, commonly deals with large amounts of data generated by nuclear magnetic resonance (NMR) and/or mass spectrometry (MS) (Ren et al., 2015). A series of procedures are involved in metabolomics, such as sample preparation, sample analysis (LC-MS, GC/MS, or NMR), data acquisition, analysis, and interpretation. In sample analysis, liquid chromatography-mass spectrometry (LC-MS) and LC-high resolution (HR-MS) are frequently utilized (El-Hawary et al., 2021). Herein, Saudi Tamarix honey was prepared and analyzed in positive and negative ion mode by LC-HRMS; a total of 336 features were detected, 299 in positive mode and 37 in negative mode. Untargeted metabolomic approaches were performed to profile metabolites in Saudi Tamarix honey considering only low molecular weight (*m/z* < 1500) ionizable molecules. Dereplication was implemented using online databases and literature reviews, the resulted features were reduced by applying a chemo-taxonomic filter, which resulted in the identification of 15 metabolites. The identified metabolites were mainly represented by phenolic acids, flavonoids, sesquiterpene and sterols, where phenols and phenolic acids derivatives were found to predominate. From literature database (Table 1), the mass ion peaks at *m/z* 359.1247 [M-H]^+^ (RT, 2.569 min), 167.0123 [M-H]^+^ (RT, 2.056 min), 179.0585 [M- H]^_^ (RT, 2.657 min), 183.08 [M ^_^ H]^+^ (RT, 22.66 min), 195.1021 [M-H]^+^ (RT, 23.56 min), and 215.0357 [M-H]- (RT, 2.435 min) for the suggested molecular formulas C_9_H_10_O_2_, C_9_H_10_O_3_, C_9_H_8_O_4_, C_10_H_14_O_3_, C_10_H_10_O_4_ and C_9_H_12_O_6_ was identified as 2-methoxy-4-vinylphenol, apocynin, caffeic acid, 3,4,5-trimethoxytoluene and 4-methoxygallic acid methyl ester, respectively, they were previously obtained from *Tamarix* species (Xiao et al., 2013; Mahfoudhi et al., 2014). 2-Methoxy-4-vinylphenol was reported as a potent natural anti-inflammatory agent (Jeong et al., 2011; Asami et al., 2023). Apocynin, caffeic acid, and isoferulic acid were reported as a potent antioxidants (Savla et al., 2021; Pavlíková, 2022; Liu et al., 2024), in addition, apocynin showed NADPH oxidase (NOX) inhibitory activity especially against neural diseases (Savla et al., 2021). Moreover, the mass ion peak at *m/z* 245.0779 [M-H]^+^ (RT, 20.73 min), corresponding to the proposed molecular formula C_15_H_26_O, was identified as nerolidol, which is a naturally occurring sesquiterpene abundant in tamarix flowers and nectar, it was earlier obtained from *Tamarix boveana* and showed antioxidant and antimicrobial activities (Saidana et al., 2008; Chan et al., 2016). Whereas the mass ion peaks at *m/z* 269.0907 [M-H]^_^ (RT, 2.849 min), 285.1255 [M-H]^+^ (RT, 9.615 min), 419.3144 [M-H]^+^ (RT, 39.26 min) and 373.1463 [M-H]^+^ (RT, 11.61 min) corresponding to the molecular formulas C_15_H_10_O_5_, C_16_H_12_O_5_, C_21_H_22_O_9_, and C_20_H_20_O_7_ were suggested to be the flavonoids, apigenin, glycitein, gardenin A, and tangeretin, respectively, which are previously isolated from *Tamarix* species and showed potent antioxidant activity (Singh et al., 2024; Jiang and Huang, 2025). The metabolites, namely, avenasterol and stigmasterol with the molecular formulas C_29_H_48_O were also dereplicated from the mass ion peaks at *m/z* 413.3178 [M^_^ H]^+^ (RT, 34.55 min) and 413.3217 [M^_^H]^+^ (RT, 35.26min), respectively, these metabolites are sterols previously reported from *T. gallica* (Andhiwal et al., 1982) and they showed antioxidant and anti-inflammatory properties (Bakrim et al., 2022; Nurkolis et al., 2024). Finally, the mass ion peak at *m/z* 353.2181 [M^_^ H]^_^ (RT, 29.37 min) for the predicted molecular formula C_16_H_18_O_9_, was distinguished as chlorogenic acid, which was isolated before from *Tamarix africana* (Chekroun-Bechlaghem et al., 2019).

Since ancient times, honey has been used for its medicinal, nutritional, and sensory qualities. These qualities concern its chemical and physical metabolites (Becerril-sánchez et al., 2021). For instance, phenolic metabolites are chemicals that can be used as biomarkers of floral and geographic origin and possess antioxidant activity and sensory qualities. Our metabolomics study revealed the abundance of phenolic chemical metabolites and sterols in Saudi tamarix honey, which justify its biological activity as potent antioxidant (Alshammari et al., 2022).

## 5 Conclusion

This study demonstrates that Saudi Tamarix honey (STH) possesses promising nephroprotective effects against cisplatin-induced kidney injury in rats. STH significantly ameliorated cisplatin-induced oxidative stress by increasing antioxidant enzymes (such as SOD) and decreasing lipid peroxidation (MDA) levels. Moreover, STH effectively suppressed the production of pro-inflammatory cytokines, such as TNF-α and IL-6, which are significantly elevated in cisplatin-induced nephrotoxicity. STH inhibited the activation of caspase-3, a key executioner of apoptosis, suggesting its anti-apoptotic properties. Our results show that STH upregulates the expression of Nrf2, a key transcription factor that regulates the expression of antioxidant genes, and downregulated the expression of STAT3, a pro-inflammatory transcription factor. These findings suggest that STH exerts its nephroprotective effects through a multi-target approach, including the inhibition of oxidative stress, inflammation, and apoptosis. Metabolomics study revealed the presence of various bioactive metabolites especially phenolic acids. Moreover, numerous researches find a connection between the antioxidant activity and phenolic chemicals. This study provides a strong basis for future research on the nephroprotective effects of STH. Further investigations will focus on the identification of the specific bioactive metabolites in STH responsible for its nephroprotective effects. This can be achieved through more detailed phytochemical analyses and *in vitro* studies to evaluate the effects of individual metabolites on kidney cells. Future research will also investigate the underlying mechanisms of action in more detail. Further studies are needed to analyze the precise molecular mechanisms by which STH modulates Nrf2 and STAT3 signalling pathways.

## Data Availability

The data presented in the study are deposited in the NIH Common Fund’s National Metabolomics Data Repository (NMDR) – *Metabolomics Workbench* repository, available at: http://dx.doi.org/10.21228/M87C2M (study ID: ST003997; project ID: PR002502). Further inquiries can be directed to the corresponding authors.
